# Spatial distribution and determinant factors of intimate partner violence among reproductive age group women in Ethiopia: Using generalized structural equation modeling

**DOI:** 10.1371/journal.pone.0263811

**Published:** 2022-02-28

**Authors:** Biruk Shalmeno Tusa, Sewnet Adem Kebede, Adisu Birhanu Weldesenbet

**Affiliations:** 1 Department of Epidemiology and Biostatistics, Collage of Health and Medical Sciences, Haramaya University, Haramaya, Ethiopia; 2 Department of Epidemiology and Biostatistics, Institute of Public Health, College of Medicine and Health Sciences, University of Gondar, Gondar, Ethiopia; Tabriz University of Medical Sciences, ISLAMIC REPUBLIC OF IRAN

## Abstract

**Introduction:**

Intimate Partner Violence (IPV) is the most serious and pervasive yet under-recognized human rights violation in the world, particularly in Ethiopia. Hence, the objective of this study was to find the spatial distribution of IPV and its determinant factors in Ethiopia.

**Methods:**

Secondary data analysis was conducted among 2,687 reproductive age group women (15–49 years). The distribution of IPV across the country was observed by ArcGIS software. In SaTScan software, the Bernoulli model was fitted by Kulldorff methods to identify the purely spatial clusters of IPV. Besides, Generalized Structural Equation Model (GSEM) was used to determine factors associated with each domain of IPV (physical, emotional & sexual violence).

**Result:**

The spatial distribution of IPV was found to be clustered in Ethiopia with Global Moran’s I 0.09 (p < 0.001), and the highest IPV cluster was observed in Oromia (p < 0.001), Somali (p < 0.001) and SNNP (p<0.001) regions. Watching television and not having attitudes toward wife beating were negatively associated with physical violence. Being rich and nonsmoker were inversely associated with emotional violence. The odds of experiencing sexual violence were high among pregnant women and wives of uneducated husbands/partners. In addition, women’s decision-making autonomy and husband/partner drinking alcohol have positive and negative associations with all domains of IPV respectively.

**Conclusion:**

There was a significant clustering of IPV in Ethiopia and the highest IPV cluster was observed in Oromia, Somali and SNNP regions. Being rich, watching television, not having attitudes toward wife beating, women’s decision-making autonomy, and husband’s/partner’s high education and non-alcohol drinker status were negatively associated with IPV. The likelihood of experiencing IPV was also high among smokers and pregnant women. Thus, we recommend that improving the economic status of the household through social protection and empowerment of women in decision-making autonomy by education and employment and increasing community awareness about the consequences of IPV with particular emphasis on Oromia, Somali and SNNP regions is essential.

## Introduction

World Health Organization (WHO) defines Intimate Partner Violence (IPV) as acts of physical aggression, sexual coercion, psychological abuse, and controlling behaviors by current or former spouses or other intimate partners that causes physical, sexual, or psychological harm [1]. It is the most serious, and under-recognized public health problem that affects millions of women globally [2–4].

Intimate partner violence affects women’s physical and mental health through direct pathways such as injury, and indirect pathways such as chronic health problems that arise from prolonged stress [5]. It also leads to adverse outcomes of maternal and child health [6–9]. Furthermore, evidence suggests that IPV increases the risk of a woman committing suicide [10], and is also responsible for the increased risk of contracting HIV and AIDS-related death, [11, 12]. Victims of IPV commonly experience psychological, physical, economic, and social consequences such as depression, anxiety, sexual addiction, post-traumatic stress disorder, and substance abuse [13].

Intimate partner violence is more prevalent in developing countries compared to developed countries. Globally, nearly one-third (30%) of all women who have been in a relationship have experienced physical and/or sexual violence by their intimate partners. The WHO report showed that this prevalence was high in Africa, Eastern Mediterranean, and South-East Asia regions (37%) and lowest in the high-income regions (23%) [14].

In Ethiopia, the prevalence of lifetime IPV ranges from 20% to 78% with poor women’s empowerment and lower educational level being the primary contributing factors [15, 16]. Even though the country ratified many of the international and continental agreements that promote and protect women’s right, around 63% of women and 28% of men agree that a husband is justified in beating his wife [17]. This makes IPV not only a deep-rooted problem but also somehow acknowledged rather than challenged. In addition, there is an imbalance between men and women in terms of institutionalized gender roles and structural power [18].

Different studies were conducted in different parts of Ethiopia using a univariate analysis to determine factors associated with IPV, and low educational level and poor women’s empowerment occurred to be primary determinants of IPV [19–25]. In those studies, only one dependent variable was allowed, and the effect of multiple latent variables could not be estimated.

Generalized Structural Equation Modeling (GSEM) is the best strategy to fill the existing gap by considering the three domains (physical, emotional and sexual violence) of IPV as independent variables. Furthermore, the prevalence of IPV is geographically not homogenous [15, 17]. Therefore the present study applies GSEM to determine factors associated with the three domains of IPV and spatial analysis to identify the geographic distribution of IPV.

## Methods and materials

### Study design and setting

Secondary data analysis was conducted on the Ethiopian Demography and Health Surveys (EDHS) 2016. Ethiopia is composed of 9 national regional states, namely Tigray, Afar, Amhara, Oromia, Somali, Benishangul-Gumuz, Southern Nations Nationalities, and Peoples’ Region (SNNPR), Gambella and Harari, and two administrative cities (Addis Ababa city administration and Dire Dawa city council). The country has 68 zones, 817 districts, and 16,253 kebeles (smallest administrative units of a country) [17]. Its current population is 114,708,673 as of Tuesday, June 2, 2020, based on Worldometer elaboration of the latest United Nations data [26].

#### Data source and study period

The data source for this study is secondary data, which was retrieved from the DHS program official database www.measuredhs.com after permission was granted as a result of an online request made by explaining the objective of our study. The 2016 Ethiopian Demographic and Health Survey (EDHS) is the fourth Demographic and Health Survey conducted in Ethiopia. The study period for the EDHS 2016 was from January 18, 2016, to June 27, 2016 [17].

### Sampling procedure, study population and sample size

The 2016 EDHS sample was stratified and selected in two stages. In the first stage, a total of 645 Enumeration Areas (EAs) (202 in urban areas and 443 in rural areas) were selected with probability proportional to EA size and with independent selection in each sampling stratum.

In the second stage of selection, a fixed number of 28 households per cluster were selected with an equal probability of systematic selection from the newly created household listing [17]. In total, 15,683 women, who were 15–49 years of age and who reported ever being married participated in the survey for the domestic violence module, only one married woman per household was selected and a total of 2,687 women were selected and interviewed. The current study included women who reported ever being married and completed the IPV questionnaire (weighted sample = 2,734). Latitude and longitude coordinates were also taken from selected enumeration areas (clusters). The detailed sampling procedure was presented in the full EDHS report [17].

### Measurements of the outcome variable and operational definition

The outcome variables with important predictors were extracted from the Ethiopian Demographic and Health Surveys individual data set. In the 2016 EDHS, IPV was assessed using women’s self-reported responses to questions depending on the modified Conflict Tactic Scales of Straus [27]. All ever-married women aged 15–49 years were selected and interviewed for the domestic violence module. Specifically, violence committed by current husbands/partners on currently married women and by the most recent husbands/partners on formerly married women was measured by asking all ever-married women if their husbands/partners ever did the following:

1. **Physical violence**: push you; shake you; or throw something at you; slap you; twist your arm or pull your hair; punch you with his/her fist or with something that could hurt you; kick you, drag you, or beat you up; try to choke you or burn you on purpose; or threaten or attack you with a knife, gun, or any other weapon. The respondents were categorized as having lifetime physical violence if they reported at least one act since the age of 15 years [17].
2. **Emotional violence**: say or do something to humiliate you in front of others; threaten to hurt or harm you or someone close to you; insult you or make you feel bad about yourself. The respondents were categorized as having lifetime emotional spousal violence if they reported at least one act since the age of 15 years [17].
3. **Sexual violence**: physically force you to have sexual intercourse with him even when you did not want to; physically force you to perform any other sexual acts you did not want to; force you with threats or in any other way to perform sexual acts you did not want to. The respondents were categorized as having lifetime sexual violence if they reported at least one act since the age of 15 years [17].
4. **Intimate partner violence (IPV)**: respondents were classified as having experienced lifetime IPV if they said that they have experienced at least one event of physical or emotional or sexual violence since the age of 15 years [17].

### Measurements of explanatory variable and operational definition

**Fig 1:**
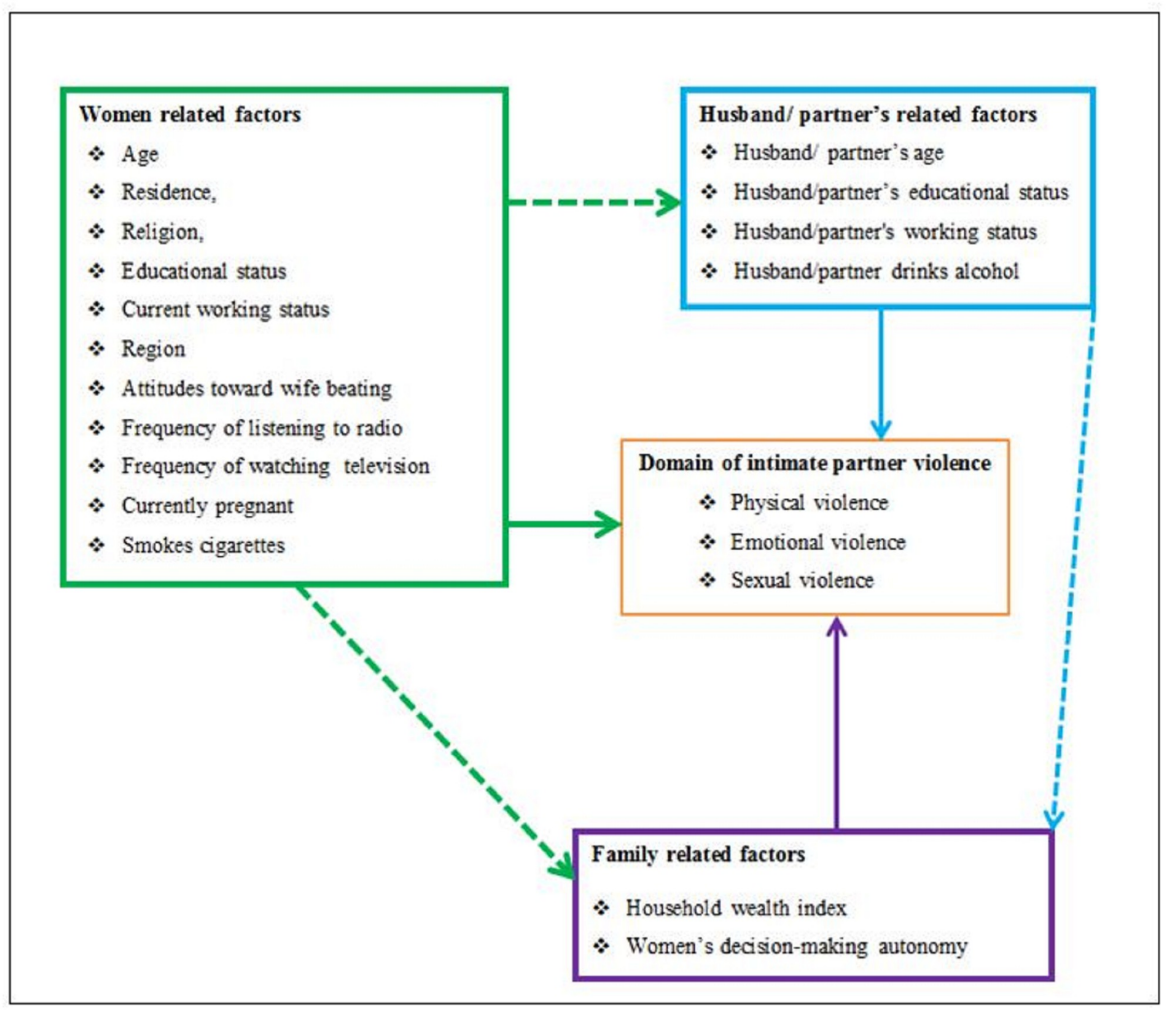
Hypothesized model for spatial distribution and determinant factors of intimate partner violence among reproductive age group women in Ethiopia.

Depending on the different works of literature reviewed [28–41]; variables related to women (age, place of residence, religion, educational status, current working status, attitudes toward wife beating, region, frequency of watching television, frequency of listening to radio, cigarette smoking and current pregnancy) husband/ partner (husband’s/partner’s age, education level, alcohol drinking, education level, and working status) and family (household wealth index and women’s decision-making autonomy) were included in this analysis (Fig 1).

1. **Attitudes toward wife beating**: were measured based on the following five questions that women were asked about whether situations of hitting or beating a wife are justifiable: if she goes out without telling him; neglects their children; argues with him; refuses to have sex with him; and burns the food. If they said ‘yes’ to any one of the above questions, they were classified as having attitude towards wife beating.
2. **Women’s decision-making autonomy**: was categorized as ‘yes’ if a woman was involved in all decisions regarding her own health care, major household purchases, and visits to her family or relatives.

### Data processing

The analysis was done using STATA 14, ArcGIS 10.3, and SaTScan 9.6 software. Before any statistical analysis, the data were weighted using sampling weight (weight for domestic violence), primary sampling unit, and strata to restore the representativeness of the survey and to tell the STATA to consider the sampling design when calculating standard errors to get reliable statistical estimates. Descriptive statistics and summary statistics were shown using text and tables.

### Spatial analysis

#### Spatial autocorrelation analysis

To check whether IPV was spread or cluster or randomly distributed, spatial autocorrelation (Global Moran’s I) statistic measure was used. Moran’s I values close to -1 indicated disease/event dispersed, whereas Moran’s I values close to +1 indicated disease/event clustered, and disease/event distributed randomly was explained by zero Moran’s I value. A statistically significant Moran’s I (p < 0.05) led to the rejection of the null hypothesis (IPV is randomly distributed) and indicated the presence of spatial autocorrelation. This analysis was done using ArcGIS software.

#### Spatial scan statistical analysis

Statistically significant spatial clusters of IPV among reproductive age group women were identified by spatial scan statistical analysis using Kuldorff’s SaTScan software. The maximum cluster size was set at 50% of the population at risk. Bernoulli’s model was fitted by considering women who did not experience lifetime IPV as controls and those women who experience lifetime IPV as cases represented by a 0/1 variable. Primary and secondary clusters were identified by using p-values and likelihood ratio tests based on the 999 Monte Carlo replications.

### Model building for Generalized Structural Equation model

Generalized Structural Equation Model (GSEM) was used to determine factors associated with each domain of IPV (physical, emotional, and social). Each domain of IPV was a binary variable that was analyzed with the Bernoulli family and a logit link function.

The analysis was started with a hypothesized model in Fig 1. Modifications were taken iteratively by adding a path link. In the end, an over-identified model with minimum information criteria was retained. A final model was selected based on the statistical significance of path coefficient, the theoretical meaningfulness of the relationship, and minimum information criteria. Statistically significant effects were considered for P < 0.05 at a confidence interval of 95%.

## Result

### Characteristics of the study population

Data from 2,687 reproductive age group women were included in the final analysis. Among these study participants, 1,083 (39.62%) were from the Oromia region, 851 (68.87%) of them were not working and 1,107 (41.20%) were Muslims. More than three-quarters (78.5%) of the respondents were not watching television at all. Around 11.1% of the respondents were pregnant. Two thousand one hundred fifty-six (69.00%) women had a decision-making autonomy, and 1,876 (68.63) women had an attitude towards wife-beating. Regarding the husband /partner of respondents, 44.80% of them did not have any formal education, and 30.23% of them drunk alcohol (Table 1).

**Table 1:** Socio-demographic characteristics of respondents in Ethiopia from January 18 to June 27, 2016 (N = 2,687).

Variables	Weighted frequency	Percent%
**Age**		
15–19	137	5.02
20–24	453	16.56
25–29	686	25.09
30–34	577	21.11
35–39	464	16.97
40–44	283	10.36
45–49	134	4.88
**Residence**		
Urban	387	14.15
Rural	2,347	85.85
**Region**		
Tigray	159	5.80
Afar	16	0.57
Amhara	687	25.14
Oromia	1,083	39.62
Somali	62	2.28
Benishangul	30	1.09
SNNPR	596	21.79
Gambella	6	0.21
Harari	5	0.18
Addis Ababa	78	2.86
Dire Dawa	12	0.45
**Religion**		
Orthodox	1,032	38.41
Muslim	1,107	41.20
Protestant	501	18.65
Traditional	19	0.71
Catholic	14	0.52
Other	14	0.52
**Educational status**		
No education	1,707	62.43
Primary	762	27.86
Secondary	168	6.16
Higher	97	3.55
**Currently working**		
Yes	851	31.13
No	1,883	68.87
**Smokes cigarettes**		
Yes	2,718	99.41
No	16	0.59
**Attitudes toward wife beating**		
Have	1,876	68.63
Have no	858	31.37
**Frequency of listening to radio**		
Not at all	1,949	71.30
Less than once a week	387	14.16
At least once a week	398	14.54
**Frequency of watching television**		
Not at all	2,156	78.85
Less than once a week	275	10.07
At least once a week	303	11.08
**Currently pregnant**		
Yes	301	11.01
No or unsure	2,433	88.99
**Household wealth index**		
Poorest	483	17.68
Poorer	584	21.37
Middle	574	20.98
Richer	544	19.91
Richest	549	20.06
**Women’s decision-making autonomy**		
Yes	1,890	69.00
No	844	31.00
**Husband/partner age**		
15–19	13	0.47
20–24	122	4.47
25–29	434	15.88
30–34	565	20.65
35–39	473	17.29
40–44	466	17.06
45–49	344	12.59
50–54	204	7.45
>55	114	4.13
**Husband/partner’s education level**		
No education	1,225	44.80
Primary	1,075	39.34
Secondary	229	8.37
Higher	190	6.94
Don’t know	15	0.55
**Husband/partner’s working status**		
Yes	2,583	94.49
No	151	5.51
**Husband/partner drinks alcohol**		
Yes	826	30.23
No	1,908	69.77

SNNPR: Southern Nation and Nationality and Peoples Region.

### Spatial distribution of intimate partner violence

**Fig 2:**
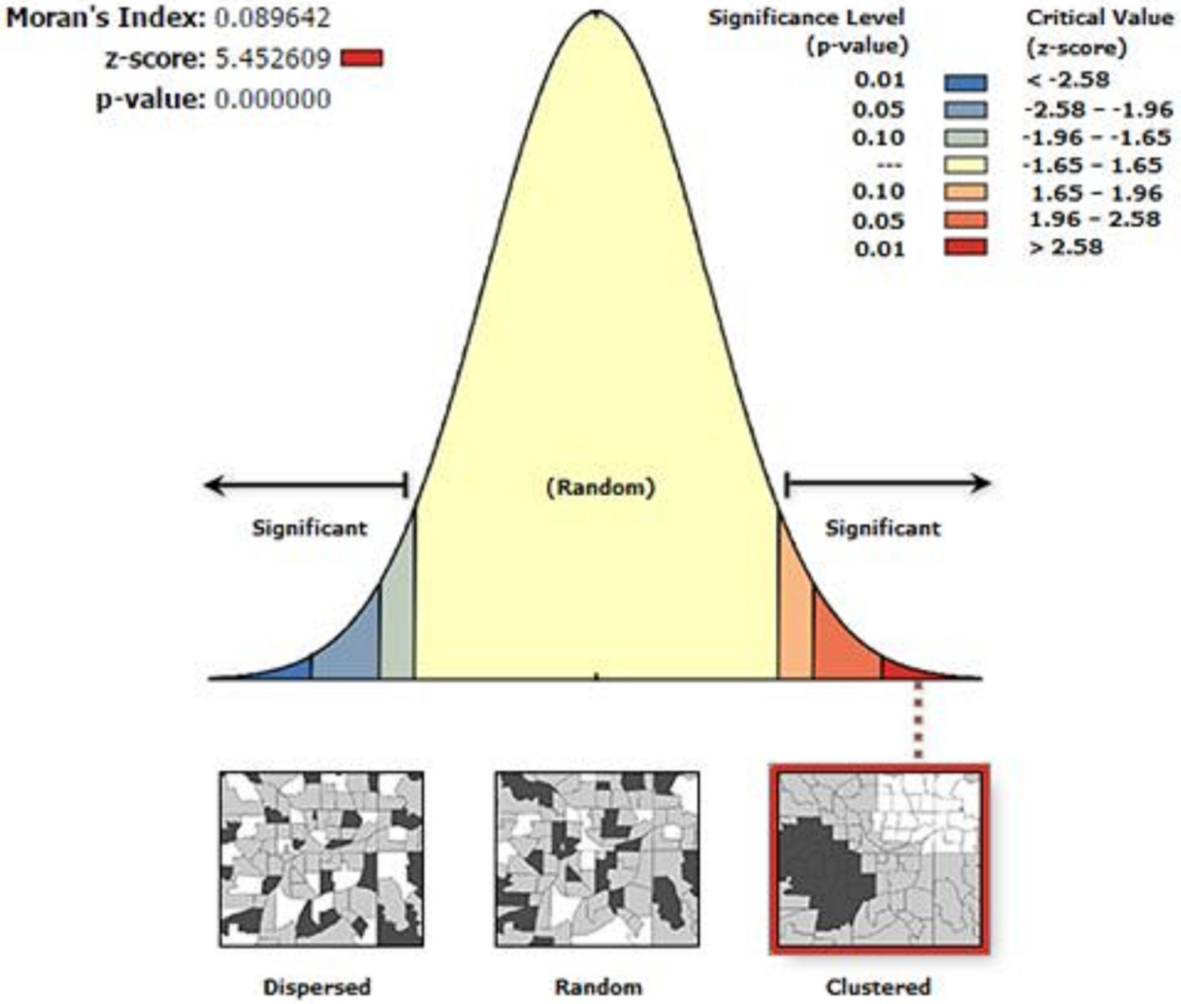
Spatial autocorrelation analysis of intimate partner violence in Ethiopia, 2016.

**Fig 3:**
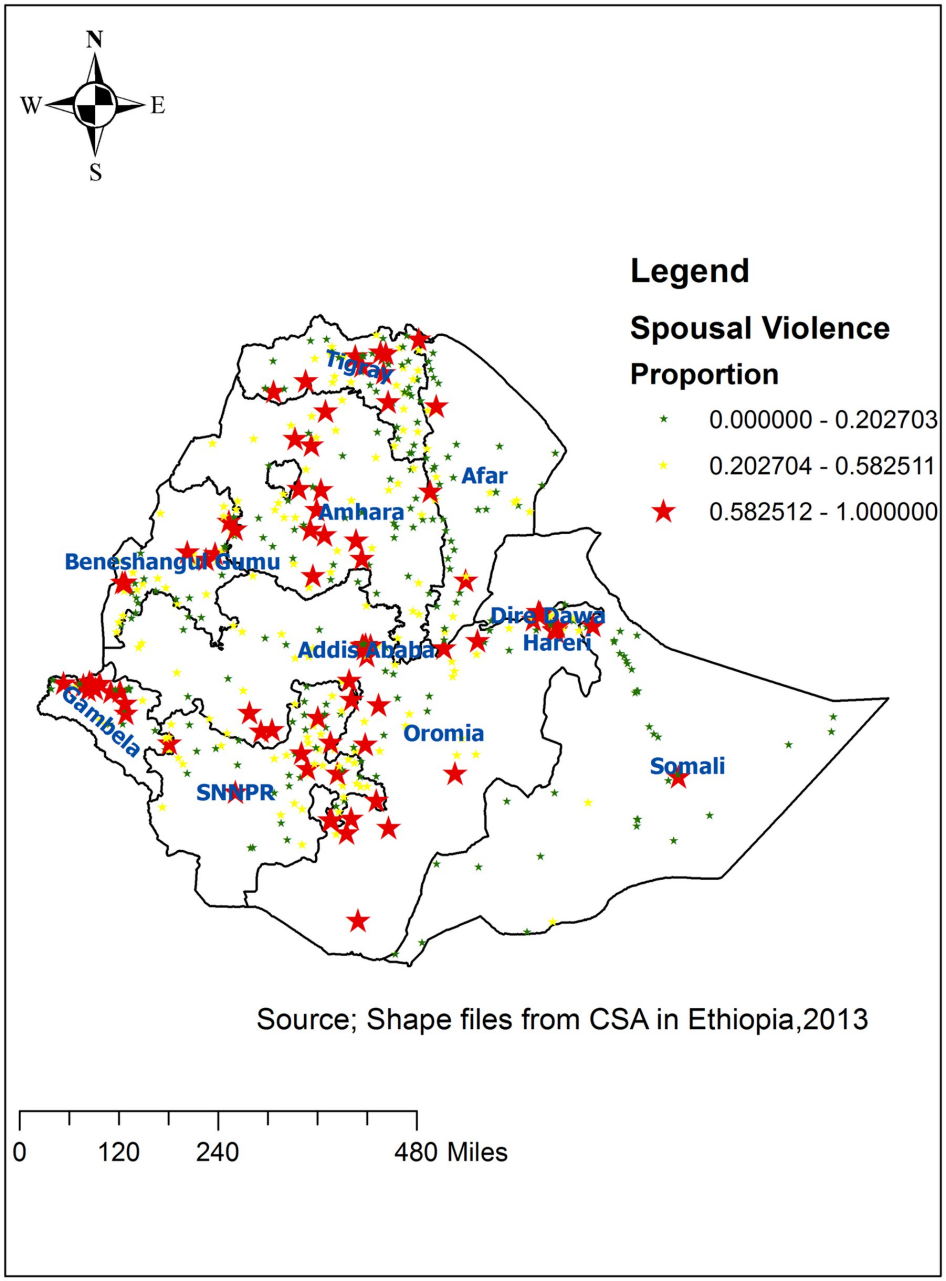
Spatial distribution of intimate partner violence across regions in Ethiopia, 2016.

The spatial distribution of IPV was found to be clustered in Ethiopia with Global Moran’s I 0.09 (p < 0.001). Given the z-score of 5.45, there is less than a 1% likelihood that this clustered pattern could be the result of random chance. The bright red and blue colors to the end tails show an increased significance level (Fig 2). Accordingly, spatial clustering of IPV was found at regional levels. The highest IPV was spatially clustered in Oromia, Amhara, SNNP and Tigray regions (Fig 3).

### Spatial SaTScan analysis of intimate partner violence (Bernoulli based model)

**Fig 4:**
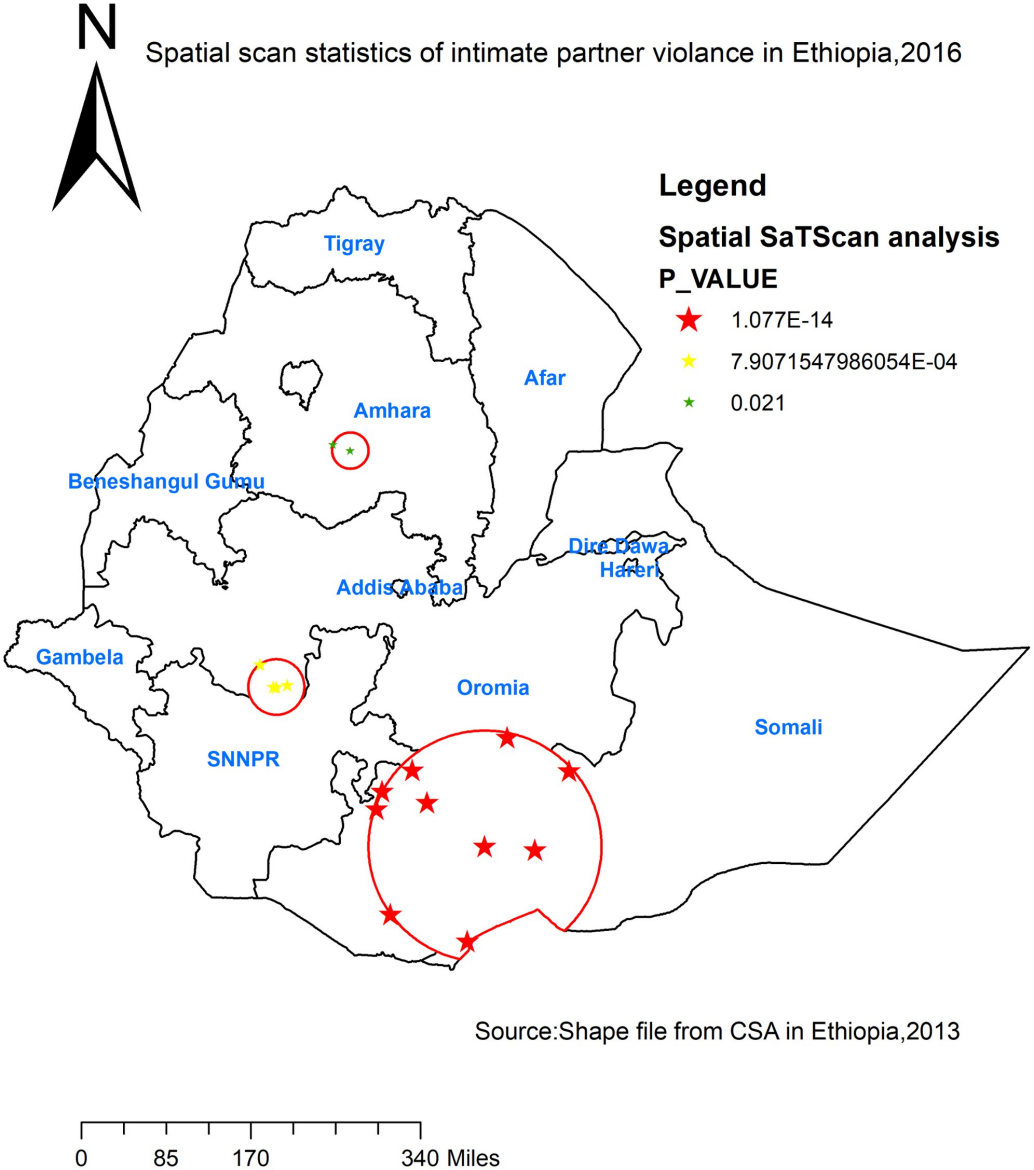
Primary and secondary clusters of health insurance coverage among women across regions in Ethiopia, 2016.

Spatial scan statistics detected a total of high and modest performing spatial clusters of IPV. Among these, 10 clusters were high-performing clusters (LLR = 40.67, RR = 2.46, P-value<0.001) and 4 clusters were the low performing clusters (LLR = 13.19, RR = 1.98, P-value<0.001). The high-performing clusters of IPV were identified in Oromia, Somali, and SNNPR (Table 2). The bright red colors (rings) indicate that the most statistically significant spatial windows of IPV. There was high IPV within the cluster than outside the cluster (Fig 4).

**Table 2:** Significant spatial clusters with high rate intimate partner violence among women in Ethiopia, 2016.

Cluster	Enumeration area (cluster) identified	Coordinate (radius)	Population	Case	RR	LLR	P-value
1	377, 394, 422, 7, 34, 289, 480, 398, 316, 601	(5.203234 N, 40.019732 E) / 187.83 km	99	75	2.46	40.67	< 0.001
2	447, 486, 227, 432	(7.527086 N, 36.970948 E) / 45.13 km	67	42	1.98	13.19	< 0.001

LLR: Likelihood ratio; RR: Relative risk.

### Determinant factors of intimate partner violence

**Fig 5:**
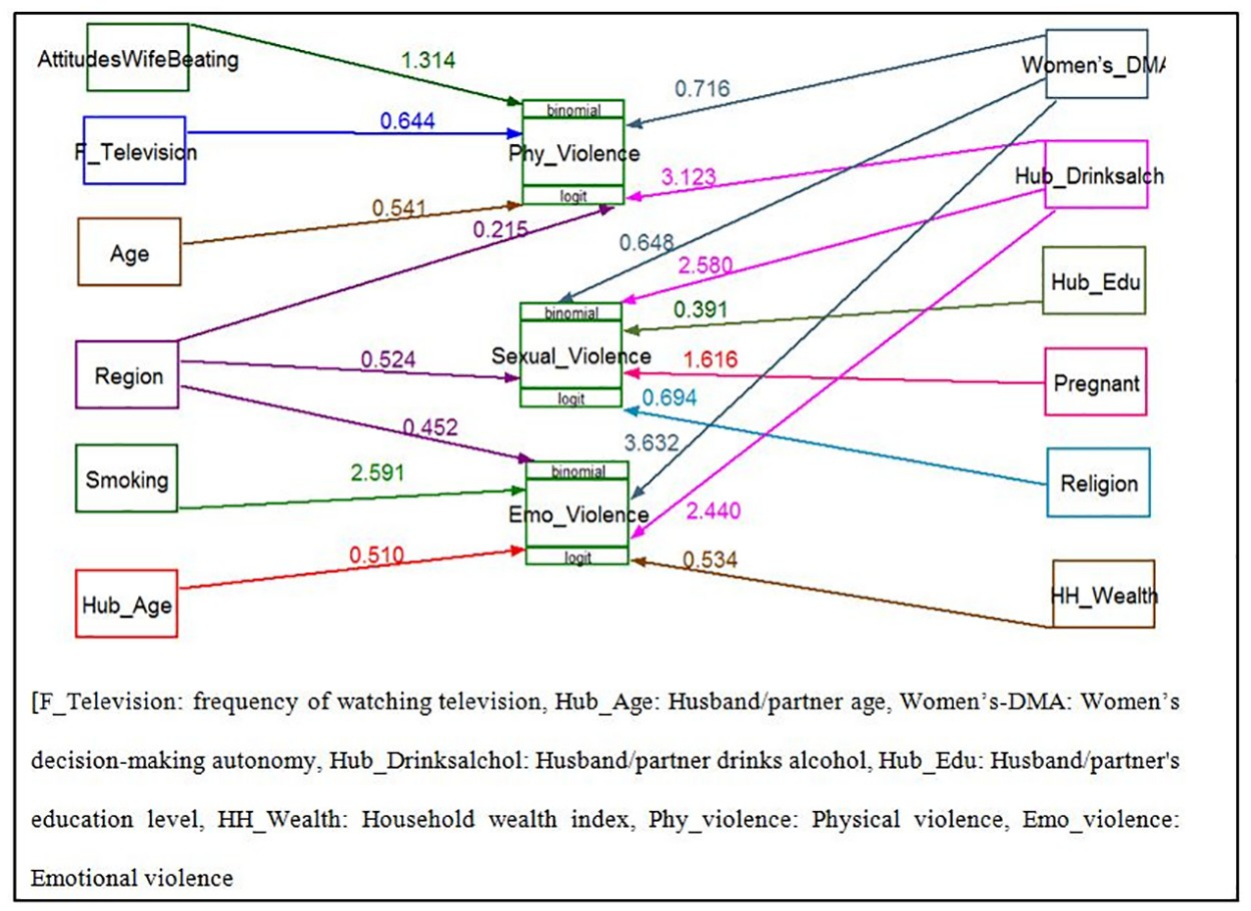
GSEM for determinant factors of intimate partner violence among reproductive age group women in Ethiopia, 2016.

The final model for determinant factors of IPV is shown in Fig 5 and Table 3. This model had included twelve exogenous variables (age, religion, region, wealth index, frequency of watching television, cigarette smoking, current pregnancy, attitudes toward wife beating, women’s decision-making autonomy, husband’s/partner’s age, husband’s/partner’s education level, husband/partner alcohol drinking status) and three endogenous variables (physical, emotional and sexual violence). All path coefficients were statistically significant at an alpha level of 0.05.

**Table 3:** Determinant factors of intimate partner violence among reproductive age group women in Ethiopia, 2016.

Variables	Physical violence		Emotional Violence		Sexual Violence	
	AOR	95% CI	AOR	95% CI	AOR	95% CI
**Age**						
25–29	Ref	Ref	Ref	Ref	Ref	Ref
15–19	**0.54**	**(0.31–0.93)**	-	-	-	-
20–24	0.97	(0.71–1.32)	-	-	-	-
30–34	1.06	(0.80–1.43)	-	-	-	-
35–39	1.30	(0.95–1.77)	-	-	-	-
40–44	1.06	(0.71–1.57)	-	-	-	-
45–49	1.04	(0.62–1.76)	-	-	-	-
**Religion**						
Muslim	Ref	Ref	Ref	Ref	Ref	Ref
Orthodox	-	-	-	-	0.88	(0.54–1.42)
Protestant	-	-	-	-	0.85	(0.51–1.41)
Traditional	-	-	-	-	**3.64**	**(1.19–11.15)**
Catholic	-	-	-	-	0.66	(0.08–5.44)
**Region**						
Oromia	Ref	Ref	Ref	Ref	Ref	Ref
Tigray	**0.22**	**(0.13–0.34)**	0.71	(0.48–1.06)	**0.52**	**(0.29–0.94)**
Afar	**0.36**	**(0.21–0.62)**	**0.46**	**(0.26–0.81)**	**0.18**	**(0.06–0.51)**
Amhara	**0.36**	**(0.25–0.52)**	**0.57**	**(0.40–0.82)**	**0.42**	**(0.25–0.71)**
Somali	**0.16**	**(0.09–0.31)**	**0.21**	**(0.11–0.38)**	**0.03**	**(0.01–0.21)**
Benishangul	**0.50**	**(0.34–0.74)**	0.85	(0.58–1.24)	**0.36**	**(0.20–0.63)**
SNNPR	**0.53**	**(0.38–0.75)**	0.80	(0.57–1.12)	**0.40**	**(0.23–0.69)**
Gambella	**0.67**	**(0.43–1.06)**	0.97	(0.62–1.53)	0.50	(0.25–1.01)
Harari	1.20	(0.76–1.89)	1.99	(1.28–3.09)	**0.42**	**(0.18–0.97)**
Addis Ababa	0.60	(0.35–1.05)	0.79	(0.46–1.36)	**0.21**	**(0.08–0.56)**
Dire Dawa	0.73	(0.46–1.16)	1.12	(0.71–1.78)	**0.37**	**(0.17–0.80)**
**Wealth index**						
Poorest	Ref	Ref	Ref	Ref	Ref	Ref
Poorer	-	-	0.91	(0.67–1.23)	-	-
Middle	-	-	1.01	(0.73–1.38)	-	-
Richer	-	-	0.87	(0.63–1.21)	-	-
Richest	-	-	**0.56**	**(0.40–0.78)**	-	-
**Frequency of watching television**						
Not at all	Ref	Ref	Ref	Ref	Ref	Ref
Less than once a week	**0.64**	**(0.45–0.93)**	-	-	-	-
At least once a week	**0.58**	**(0.41–0.83)**	-	-	-	-
**Smokes cigarettes**						
No	Ref	Ref	Ref	Ref	Ref	Ref
Yes	-	-	**2.59**	**(1.18–5.67)**	-	-
**Currently pregnant**						
No or unsure	Ref	Ref	Ref	Ref	Ref	Ref
Yes	-	-	-	-	**1.62**	**(1.06–2.46)**
**Attitudes toward wife beating**						
Have no	Ref	Ref	Ref	Ref	Ref	Ref
Have	**1.31**	**(1.05–1.65)**	-	-	-	-
**Women’s decision-making autonomy**						
No	Ref	Ref	Ref	Ref	Ref	Ref
Yes	**0.72**	**(0.58–0.89)**	**0.70**	**(0.57–0.86)**	**0.65**	**(0.47–0.89)**
**Husband/partner age**						
30–34	Ref	Ref	Ref	Ref	Ref	Ref
15–19	-	-	0.60	(0.12–3.03)	-	-
20–24	-	-	**0.51**	**(0.30–0.87)**	-	-
25–29	-	-	**0.62**	**(0.45–0.87)**	-	-
35–39	-	-	0.77	(0.57–1.05)	-	-
40–44	-	-	1.05	(0.77–1.44)	-	-
45–49	-	-	1.14	(0.81–1.61)	-	-
50–54	-	-	1.10	(0.73–1.65)	-	-
55–59	-	-	1.50	(0.90–0.50)	-	-
**Husband/partner’s education level**						
No education	Ref	Ref	Ref	Ref	Ref	Ref
Primary	-	-	-	-	1.13	(0.81–1.58)
Secondary	-	-	-	-	0.71	(0.40–1.29)
Higher	-	-	-	-	**0.39**	**(0.16–0.93)**
Don’t know	-	-	-	-	1.19	(0.26–5.42)
**Husband/partner drinks alcohol**						
No	Ref	Ref	Ref	Ref	Ref	Ref
Yes	**3.12**	**(2.45–3.97)**	**2.44**	**(1.94–3.08)**	**2.58**	**(1.75–3.80)**

Numerous variables, namely place of residence, educational status, currently working, frequency of listening to radio, husband’s/partner’s education level, husband’s/partner’s working status were excluded from the final model as their effect was not statistically significant at an alpha level of 0.05.

The likelihood of experiencing physical violence has decreased by 46% among women aged 15–19 years than women aged 25–29 years. Traditional religion followers were 3.64 times more likely to be sexually violated than Muslims. The odds of emotional violence have decreased by 44% among the richest than the poorest.

The odds of experiencing physical violence were low in Tigray, Afar, Amhara, Gambella, Benishangul, SNNP, and Somali regions as compare with the Oromia region. In addition, the likelihood of emotional violence was low in Afar, Amhara, and Somali regions when it is compared with the Oromia region. Furthermore, the chance of sexual violence was low among women from Tigray, Afar, Amhara, Somali, Benishangul, SNNPR, Somalia regions, and Harari, Addis Ababa, Dire Dawa city administrations compared to those women from the Oromia region.

The likelihood of experiencing physical violence has decreased by 36% and 42% among women who were watching television less than once a week and at least once a week as compared with those who were not watching television at all respectively. Smoker women were 2.59 times more likely to be emotionally violated than their counterparts. Besides, pregnant women were 1.62 times more probable to be sexually violated than non-pregnant women.

Women who have attitudes toward wife beating were 1.32 times more likely to be physically violated than their counterparts. The chances of physical, emotional, and sexual violence have decreased by 28%, 30%, and 35% among women who had decision-making autonomy than their counterparts respectively.

Husbands /partner’s age, educational level and alcohol drinking status were significant husband /partner-related factors that determine IPV. The likelihoods of emotional violence have decreased by 49% and 38% among husbands/partners aged 20–24 and 25–29 years as compared with husbands/partners aged 30–34 years respectively. The odd of sexual violence has decreased by 61% among highly educated husbands/partners than the uneducated. Drinker husbands /partners were 3.12, 2.44 and 2.58 times more likely to physically, emotionally and sexually violate their wives/ partners than their counterparts respectively.

## Discussion

The purpose of this study was to find the spatial distribution of IPV and its determinant factors in Ethiopia. According to the present study, there was a significant clustering of IPV in the study area, and the highest IPV cluster was observed in Oromia, Somali and SNNP regions. This clustering of IPV in these areas might be due to the culture of a given society that did not recognize IPV as a violation of human rights rather accepted and expected that wife-beating is part of a normal union. This also might be low awareness and wrong attitude of husbands/partners toward the negative consequence of women violence.

The present study documented that women’s age is a determinate factor of IPV. Relatively younger women were less likely to be physically abused as compared to older women. This finding is lined with household surveys in eight Southern African countries [29] and Rwanda [30]. This similar finding might be related to the duration of the partnership of women’s first IPV victimization. Most of the time relationships of longer durations are more likely to involve a history of male-perpetrated violence [42].

This study also revealed that women who were watching television were at low risk of physical violence as compared with their counterparts. This result might be due to the positive effect of mass media like the television on women’s attitudes towards violence against women [43] and those women who have such attitudes are less likely to be abused.

Cigarettes smoking status is significantly associated with emotional violence. According to the present study, smoker women were more likely to be emotionally violated than their counterparts. Since the current study used secondary data that was conducted through community-based cross-sectional study, it was difficult to sort out the causal ordering between cigarette smoking status and emotional violence. Some studies also documented that experiencing intimate partner violence (IPV) increases women’s risk for cigarette smoking [44–47].

The results of this study suggested that pregnant women were more probable to be sexually violated than non-pregnant women. Such violence during pregnancy does not only affect the women’s reproductive health but also leads to fatal and non-fatal adverse health outcomes due to the direct trauma of abuse of a pregnant woman’s body, as well as the physiological effects of stress from current or past abuse on fetal growth and development [48–50].

In line with a study conducted in Uganda [51], the present study reported that women who had attitudes toward wife beating were more likely to be physically violated than their counterparts. The possible explanation for these results is that women with attitudes supportive of IPV are more likely to be victims of IPV. Because of such attitudes, women may accept and expect that wife-beating is part of a normal union.

Our finding demonstrated that women’s decision-making autonomy was significantly associated with physical, emotional, and sexual violence. The chances of physical, emotional, and sexual violence were low among women who had decision-making autonomy than their counterparts. This finding is in line with other studies that were conducted in Uganda [41], in Ghana [37], and in Peru [36]. This consistency could be supported by a male-dominated marital power structure that has been documented to be highly related to marital conflict and husband-to-wife violence [52]. Women’s decision-making autonomy has not only reduced the risk IPV but also increased the utilization of maternal services [35, 53].

In line with other studies [33, 34], the current study reported that women with a higher wealth index have less chance of experiencing emotional violence. The possible explanation for these consistent results might be that low household economic status is the reason for conflict between couples.

The current study also identified husband /partner-related factors that are associated with IPV. The factors included husbands /partner’s age, educational level, and alcohol drinking status. The present study reported that the likelihoods of emotional violence were low among young husbands/partners as compared with old husbands/partners. A similar result has been found in Haiti [30]. Concurrent with other studies [31, 32], the present study reported that the likelihoods of sexual violence were low among highly educated husbands/partners than the uneducated.

This study revealed that a husband/partner who drinks alcohol is a predictor of IPV. According to the current study, drinker husbands /partners were more likely to physically and sexually violate their wives/ partners than their counterparts. This result is in line with other studies that were conducted in Angola [40], Uganda [38], and India [39]. The possible explanation for these results is that alcohol use directly disturbs mental and physical function, decreasing self-control and leaving individuals less capable of negotiating a non-violent resolution to conflicts in relationships. Excessive drinking by one partner can worsen financial problems, child care difficulties, infidelity or other family stressors. This can lead to marital tension and conflict, increasing the risk of violence occurring between partners.

The primary strength of the current study was using large population-based data with a large sample size, which is representative at national and regional levels, so it can be generalized to all women in the reproductive age group in Ethiopia. The joint use of ArcGIS and Sat Scan statistical tests facilitated to identify of similar and statistically significant areas with high IPV (hot spot areas). Furthermore, it used multivariate analysis (GSEM) to determine factors associated with the three domains of IVP simultaneously.

However, the finding of this study was interpreted with some limitation. First, the location of data values was displaced up to 2 kilometers for urban and up to 5 kilometers for rural areas to ensure respondent confidentiality; thus, this was a challenge to know the exact cases’ location. Since EDHS was conducted using cross-sectional study design, it was difficult to sort out the causal ordering. Recall bias may be the other impediment for this study as EDHS was a questionnaire-based survey and relied on the memory of the respondents.

## Conclusion

There was a significant clustering of IPV in Ethiopia, and the highest IPV cluster was observed in Oromia, Somali and SNNP regions. Being rich, watching television, not having attitudes toward wife beating, women’s decision-making autonomy, and husband’s/partner’s high education and non-alcohol drinker status were negatively associated with IPV. The likelihood of experiencing IPV was also high among smokers and pregnant women. Thus, we recommend that improving the economic status of the household through social protection and empowerment of women’s decision making autonomy through education and employment and increasing community awareness about the consequences of IPV with particular emphasis on Oromia, Somali and SNNP regions is important.

## Abbreviations

CSA: Central Statistics Agency
EAs: Enumeration Areas
EDHS: Ethiopian Demographic and Health Survey
GSEM: Generalized Structural Equation Model
IPV: Intimate partner violence
SNNPR: Southern Nation and Nationality and Peoples Region
WHO: World Health Organization
